# Genetic Diversity and Population Structure of *Leishmania infantum* from Southeastern France: Evaluation Using Multi-Locus Microsatellite Typing

**DOI:** 10.1371/journal.pntd.0004303

**Published:** 2016-01-25

**Authors:** Christelle Pomares, Pierre Marty, Anne Laure Bañuls, Emmanuel Lemichez, Francine Pratlong, Benoît Faucher, Fakhri Jeddi, Sandy Moore, Grégory Michel, Srikanth Aluru, Renaud Piarroux, Mallorie Hide

**Affiliations:** 1 INSERM, U1065, Centre Méditerranéen de Médecine Moléculaire, C3M, Toxines Microbiennes dans la Relation Hôte–Pathogènes, Nice, France; 2 Université de Nice Sophia Antipolis, Faculté de Médecine, Nice, France; 3 Parasitologie-Mycologie, Centre Hospitalier Universitaire l’Archet, CS 23079 06202, Nice, France; 4 UMR MIVEGEC IRD 224-CNRS 5290-Université Montpellier, Montpellier, France; 5 Département de Parasitologie–Mycologie, CHRU de Montpellier, Centre National de Référence des Leishmanioses, Montpellier, France; 6 Service des Maladies Infectieuses, CHU Hôpital Nord, Assistance Publique-Hôpitaux de Marseille, Marseille, France; 7 Aix-Marseille Université, UMR MD3, Marseille, France; 8 Aix–Marseille Université, Marseille, France; Charité University Medicine Berlin, GERMANY

## Abstract

In the south of France, *Leishmania infantum* is responsible for numerous cases of canine leishmaniasis (CanL), sporadic cases of human visceral leishmaniasis (VL) and rare cases of cutaneous and muco-cutaneous leishmaniasis (CL and MCL, respectively). Several endemic areas have been clearly identified in the south of France including the Pyrénées-Orientales, Cévennes (CE), Provence (P), Alpes-Maritimes (AM) and Corsica (CO). Within these endemic areas, the two cities of Nice (AM) and Marseille (P), which are located 150 km apart, and their surroundings, concentrate the greatest number of French autochthonous leishmaniasis cases. In this study, 270 *L*. *infantum* isolates from an extended time period (1978–2011) from four endemic areas, AM, P, CE and CO, were assessed using Multi-Locus Microsatellite Typing (MLMT). MLMT revealed a total of 121 different genotypes with 91 unique genotypes and 30 repeated genotypes. Substantial genetic diversity was found with a strong genetic differentiation between the *Leishmania* populations from AM and P. However, exchanges were observed between these two endemic areas in which it seems that strains spread from AM to P. The genetic differentiations in these areas suggest strong epidemiological structuring. A model-based analysis using STRUCTURE revealed two main populations: population A (consisting of samples primarily from the P and AM endemic areas with MON-1 and non-MON-1 strains) and population B consisting of only MON-1 strains essentially from the AM endemic area. For four patients, we observed several isolates from different biological samples which provided insight into disease relapse and re-infection. These findings shed light on the transmission dynamics of parasites in humans. However, further data are required to confirm this hypothesis based on a limited sample set. This study represents the most extensive population analysis of *L*. *infantum* strains using MLMT conducted in France.

## Author Summary

In the south of France, the parasite *Leishmania infantum* is responsible for diseases that primarily affect dogs but can also impact humans. Several endemic areas have been clearly identified in the south of France including the Pyrénées-Orientales, Cévennes (CE), Provence (P), Alpes-Maritimes (AM) and Corsica (CO). In this study, 270 *L*. *infantum* isolates from four endemic areas, AM, P, CE and CO, were assessed using Multi-Locus Microsatellite Typing (MLMT), a tool applied for population genetic studies. MLMT revealed a strong genetic differentiation between the *Leishmania* populations from AM and P with exchanges observed between these two endemic areas. For four patients, the occurrence of disease relapses and re-infections was examined. These findings shed light on the transmission dynamics of parasites in humans. This study represents the most extensive population analysis of *L*. *infantum* isolates using MLMT conducted in France.

## Introduction

Leishmaniases are a group of diseases caused by obligatory intracellular protozoan parasites of the genus *Leishmania*. Among the species of *Leishmania*, *Leishmania infantum* is mostly responsible for canine leishmaniasis (CanL), although it also causes sporadic cases of human visceral leishmaniasis (VL), and rare cases of cutaneous and muco-cutaneous leishmaniasis (CL and MCL) throughout the Mediterranean basin [1]. Transmission to humans is caused by the bite of infected phlebotomine sandflies, and dogs are considered to be the principal domestic reservoir. In France, the parasite is currently only endemic in the south of France, along the Mediterranean coast, where several foci have been clearly identified: Pyrénées-Orientales, Cévennes (CE), Provence (P), Alpes-Maritimes (AM) and Corsica (CO) [2]. In the Provence-Alpes-Côtes d’Azur (PACA) region, which comprises the AM and P endemic areas, transmission has been reported for 100 years [3,4]. The two cities of Nice and Marseille, which are located 150 km apart, and their surroundings concentrate the greatest number of French autochthonous leishmaniasis cases [2,5]. Although the same species of *L*. *infantum* (primarily zymodeme MON-1), the same predominant vector (*Phlebotomus perniciosus*) and the same and unique reservoir (dog) are found in both regions, the transmission environment of VL is heterogeneous in these two foci [5]. Disease transmission in Nice and the surrounding area is associated with scattered habitation and mixed forest in the foothills [5]. In contrast, around Marseille, VL transmission is associated with an urban environment [5]. Regarding the main vector; *Phlebotomus pernicious*; the population is quite homogeneous and belongs mainly to the same haplogroup (for 88% pern01) in Provence, France [6]. The isolates of *L*. *infantum* from AM and P endemic foci have been characterized using Multi-Locus Enzyme Electrophoresis (MLEE), which is the current reference method. However, MLEE based analyses are limited at the intrinsic level of polymorphisms. Thus, differentiating between isolates in PACA region is impossible using the MLEE method [7]. Epidemiological studies on *L*. *infantum* require the use of highly discriminative techniques that can differentiate between MON-1 strains. Multi-Locus Microsatellite Typing (MLMT) has been shown to be a powerful tool for population genetics and epidemiological studies of *Leishmania spp*. [8]. This tool has been already applied to genotype *L*. *infantum* isolates from healthy blood donors, sandflies, dogs and human patients in Southern France [9]. Genetic differentiations were evidenced between asymptomatic carrier strains and non-asymptomatic carrier strains and especially between asymptomatic carrier and HIV+ populations [9]. However, due to the weak sample size, these results must be confirmed on a larger sample set [9].

In the current study, microsatellite markers were used to analyze the genetic diversity of *L*. *infantum* parasites from Southeast France, with a focus on the PACA region. We assessed an extensive panel of isolates from an extended time period (1978–2011), from the two endemic regions of AM and P. The geographical and temporal distributions of genotypes were examined. The microsatellite profiles were used to assess relapse and re-infection among patients as well as the association between genotype and the various clinical forms of the disease.

## Materials and Methods

### Parasites

The *L*. *infantum* isolates used in this study were obtained from the collection of the Centre National de Référence des *Leishmania* (*Leishmania* collection, BRC-Leish, Montpellier, France, BioBank N° BB-0033-00052). All human and animal samples had been isolated from patients and animals as part of routine diagnosis and treatment with no unnecessary invasive procedures. A total of 270 *L*. *infantum* isolates from the south of France were included in this study (Table 1). This panel included 247 human isolates from 239 patients (four patients harbored more than one isolate), 20 from CanL, one from feline leishmaniasis and two from sandflies. Among the 239 patients, there were 154 adult VL cases, 58 infant VL cases, 13 CL cases (five infants, seven adults and one unknown), three MCL adult cases, nine asymptomatic carriers (in adults) and two unknown cases (one adult and one unknown). Among the 247 human samples, 139 were isolated from immunocompetent patients, 95 from immunocompromised HIV+ patients, 11 from immunocompromised patients other than HIV+ (e.g., renal transplantation, lymphoma, auto immunity disease and cancer) and two from unknown cases. The location of isolates based on their position relative to the Vars River (S1 Table) was used for genetic differentiation analysis.

**Table 1:** Designation and characteristics of *Leishmania infantum* isolates used in this study.

Endemic area	Patients	Sample WHO code	Y	C F / H	HIV status	Zymo	G	P	Sub-Pop A	Sub-Pop A	Sub-Pop B
									K = 6	K = 4	K = 2
P	Patient 1	MHOM/FR/94/LPM135	1994	VL	HIV	MON-1	1	A	6	3	
AM	Patient 2	MHOM/FR/90/LPN63	1990	VL	HIV	MON-24	2	A	6	3	
AM	Patient 3	MHOM/FR/2002/LPN202	2002	IVL		MON-1	3	A	4	1	
P		MCAN/FR/2000/LPM206	2000	CanL		MON-1	4	A	4	1	
AM	Patient 4	MHOM/FR/2003/LPN212	2003	VL	HIV	MON-1	4	A	4	1	
AM	Patient 4	MHOM/FR/2004/LPN243	2004	RVL	HIV	MON-1	4	A	4	1	
AM	Patient 5	MHOM/FR/90/LPN67	1990	VL	HIV	MON-1	5	A	4	1	
AM	Patient 6	MHOM/FR/96/LPN126	1996	IVL		MON-1	6	A	4/3	1	
P	Patient 7	MHOM/FR/93/LPM110	1993	VL	HIV	MON-11	7	A	4/6	1/3	
AM	Patient 8	MHOM/FR/90/LPN62	1990	VL		MON-1	8	B			1
AM	Patient 9	MHOM/FR/98/LPN163	1998	IVL		MON-1	9	B			1
P	Patient 10	MHOM/FR/96/LPM150	1996	VL	HIV	MON-1	10	A	4/5	1/2	
AM	Patient 11	MHOM/FR/2001/LPN192	2001	IVL		MON-1	11	B/A			
CE		MCAN/FR/2006/LPN278	2006	CanL		MON-1	12	A	4/5	1/2	
P	Patient 12	MHOM/FR/97/LPM180	1997	VL	HIV	MON-1	13	A	5	2	
P		MCAN/FR/2000/LPM207	2000	CanL		MON-1	13	A	5	2	
P	Patient 13	MHOM/FR/2009/LPM262	2009	VL	HIV	MON-1	13	A	5	2	
P	Patient 14	MHOM/FR/2000/LPM197	2000	VL	HIV	MON-1	14	A	5	2	
AM	Patient 15	MHOM/FR/97/LPN161	1997	CL		MON-1	15	A	1	4	
CO	Patient 16	MHOM/FR/97/LPN154	1997	VL		MON-1	16	A	6	3	
P	Patient 17	MHOM/FR/2002/LPM225	2002	VL	HIV	MON-1	17	A	5	2	
P	Patient 18	MHOM/FR/2002/LPM215	2002	VL	HIV	MON-1	17	A	5	2	
P	Patient 19	MHOM/FR/2000/LPM 195	2000	VL	HIV	MON-1	18	A	2/5/3	1/3/2	
AM	Patient 20	MHOM/FR/86/LPN30	1986	VL	HIV	MON-1	19	A/B			
AM	Patient 21	MHOM/FR/88/LPN50	1988	VL	HIV	MON-1	20	A	5	2	
AM	Patient 29	MHOM/FR/84/LPN23	1984	VL		MON-1	21	A	5	2	
AM	Patient 30	MHOM/FR/85/LPN25a	1985	VL		MON-1	21	A	5	2	
AM	Patient 31	MHOM/FR/94/LPN112	1994	VL		MON-1	21	A	5	2	
P	Patient 22	MHOM/FR/96/LPM151	1996	VL	HIV	MON-1	21	A	5	2	
AM	Patient 32	MHOM/FR/96/LPN132	1996	VL		MON-1	21	A	5	2	
P	Patient 23	MHOM/FR/97/LPM169	1997	VL	HIV	MON-1	21	A	5	2	
P	Patient 24	MHOM/FR/97/LPM172	1997	VL	HIV	MON-1	21	A	5	2	
P	Patient 35	MHOM/FR/2004/LPN228	2004	IVL		MON-1	21	A	5	2	
P	Patient 25	MHOM/FR/2005/LPM242	2005	IVL		MON-1	21	A	5	2	
P	Patient 26	MHOM/FR/2005/LPM247	2005	IVL		MON-1	21	A	5	2	
P	Patient 27	MHOM/FR/2009/LPM261	2009	IVL		MON-1	21	A	5	2	
AM		MCAN/FR/95/LPN122*		CanL		MON-1	21	A	5	2	
AM		MCAN/FR/95/LPN123*		CanL		MON-1	21	A	5	2	
AM		MCAN/FR/95/LPN124*		CanL		MON-1	21	A	5	2	
AM	Patient 33	MHOM/FR/96/LPN136*	1996	AC		MON-1	21	A	5	2	
AM	Patient 34	MHOM/FR/96/LPN131*	1996	AC		MON-1	21	A	5	2	
AM	Patient 28	MHOM/FR/81/LPN5	1981	IVL		MON-1	21	A	5	2	
AM	Patient 36	n. d.	2009	IVL		MON-1	21	A	5	2	
AM	Patient 37	MHOM/FR/90/LPN61	1990	IVL		MON-1	22	A/B			
AM	Patient 38	MHOM/FR/2002/LPN199	2002	VL		MON-1	23	A	3	1	
P	Patient 39	MHOM/FR/2007/LPM255	2007	IVL		MON-1	24	A	4/5/2	1	
P	Patient 40	MHOM/FR/2002/LPM216	2002	VL	HIV	MON-1	25	A	4/3/2	1	
P	Patient 41	MHOM/FR/2002/LPM217	2002	VL		MON-1	25	A	4/3/2	1	
P	Patient 42	MHOM/FR/98/LPM185	1998	VL	HIV	MON-1	26	A	4/5/6	1	
CO	Patient 43	MHOM/FR/2006/LPM251	2006	IVL		MON-24	27	A	2	1	
AM	Patient 44	MHOM/FR/2001/LPN195	2001	VL		MON-1	28	A	5/2/4	1/2	
AM	Patient 45	MHOM/FR/2007/LPN312	2007	IVL		MON-1	29	A	3	1	
AM		MCAN/FR/86/LPN28	1986	CanL		MON-1	30	A	3	1	
P	Patient 46	MHOM/FR/2003/LPN221	2003	CL		MON-1	31	A	3/2	1	
AM	Patient 47	MHOM/FR/97/LPN159	1997	VL		MON-1	32	B/A			
AM	Patient 48	MHOM/FR/90/LPN64	1990	VL	HIV	MON-1	33	A	3	1	
AM	Patient 49	MHOM/FR/89/LPN54	1989	VL		MON-1	34	B			1
AM	Patient 50	MHOM/FR/91/LPN70	1991	IVL		MON-1	34	B			1
AM	Patient 51	MHOM/FR/92/LPN78	1992	VL		MON-1	34	B			1
AM	Patient 52	MHOM/FR/95/LPN116	1995	VL	HIV	MON-1	34	B			1
AM	Patient 53	MHOM/FR/96/LPN141	1996	VL		MON-1	34	B			1
AM	Patient 54	MHOM/FR/97/LPN150	1997	IVL		MON-1	34	B			1
AM	Patient 55	MHOM/FR/2001/LPN181	2001	IVL		MON-1	34	B			1
AM	Patient 56	MHOM/FR/2007/LPN313	2007	CL		MON-1	34	B			1
AM	Patient 57	MHOM/FR/2011/LPN358	2011	VL		MON-1	34	B			1
AM		MCAN/FR/89/LPN57	1989	CanL		MON-1	35	A/B			
AM	Patient 58	MHOM/FR/95/LPN120	1995	VL	HIV	MON-1	35	A/B			
AM	Patient 60	MHOM/FR/92/LPN84	1992	IVL		MON-1	36	A	3	1	
AM	Patient 61	MHOM/FR/94/LPN103	1994	CL		MON-1	36	A	3	1	
AM	Patient 62	MHOM/FR/95/LPN115	1995	IVL		MON-1	36	A	3	1	
AM	Patient 63	MHOM/FR/95/LPN119	1995	VL	HIV	MON-1	36	A	3	1	
CO	Patient 59	MHOM/FR/96/LPM157	1996	VL		MON-1	36	A	3	1	
AM	Patient 64	MHOM/FR/2001/LPN187	2001	VL		MON-1	36	A	3	1	
AM	Patient 64	MHOM/FR/2002/LPN198	2002	RVL		MON-1	36	A	3	1	
P	Patient 65	MHOM/FR/94/LPM112	1994	VL	HIV	MON-1	37	A	4/5/1	1/2/4	
P	Patient 66	MHOM/FR/2006/LPM250	2006	VL		MON-1	37	A	4/5/1	1/2/4	
AM	Patient 67	MHOM/FR/93/LPN92	1993	VL	HIV	MON-1	38	A	2/1/3	3/4/1	
P	Patient 68	MHOM/FR/97/LPM173	1997	VL		MON-1	39	B			1
AM	Patient 69	MHOM/FR/86/LPN29	1986	VL	HIV	MON-1	40	A/B			
AM	Patient 71	MHOM/FR/87/LPN33	1987	IVL		MON-1	41	A/B			
AM		MCAN/FR/87/LPN34	1987	CanL		MON-1	41	A/B			
AM	Patient 72	MHOM/FR/89/LPN51	1989	VL	HIV	MON-1	41	A/B			
AM	Patient 73	MHOM/FR/98/LPN164	1998	VL	HIV		41	A/B			
AM	Patient 70	MHOM/FR/2005/LPM244	2005	VL	HIV	MON-1	41	A/B			
AM	Patient 74	MHOM/FR/96/LPN146	1996	VL	HIV	MON-1	42	B			2
AM	Patient 75	MHOM/TR/94/LPN101	1994	IVL		MON-1	43	B			1
AM	Patient 76	MHOM/FR/94/LPN104	1994	IVL		MON-1	43	B			1
AM	Patient 77	MHOM/FR/95/LPN113	1995	IVL		MON-1	43	B			1
AM	Patient 78	MHOM/FR/95/LPN121	1995	VL	HIV	MON-1	43	B			1
AM	Patient 86	MHOM/FR/2002/LPN201	2002	RVL	HIV	MON-1	43	B			1
AM	Patient 87	MHOM/FR/2004/LPN233	2004	VL	HIV	MON-1	43	B			1
AM	Patient 79	MHOM/FR/96/LPN134*	1996	AC		MON-1	43	B			1
AM	Patient 80	MHOM/FR/96/LPN137*	1996	AC		MON-1	43	B			1
AM	Patient 81	MHOM/FR/96/LPN138*	1996	AC		MON-1	43	B			1
AM	Patient 82	MHOM/FR/96/LPN144*	1996	AC		MON-1	43	B			1
AM	Patient 83	MHOM/FR/96/LPN143*	1996	AC		MON-1	43	B			1
AM	Patient 84	MHOM/FR/96/LPN142*	1996	AC		MON-1	43	B			1
AM	Patient 85	MHOM/IT/96/LPN145*	1996	AC		MON-1	43	B			1
AM	Patient 88	MHOM/FR/80/LPN3	1980	IVL		MON-1	44	B			1
AM	Patient 89	MHOM/00/94/LPN108	1994	VL	HIV	MON-1	44	B			1
AM	Patient 90	MHOM/FR/2004/LPN236	2004	VL		MON-1	45	B			1
AM		MCAN/FR/82/LPN16	1983	CanL		MON-1	46	B			1
AM	Patient 91	MHOM/FR/83/LPN19	1983	VL		MON-1	46	B			1
AM	Patient 92	MHOM/FR/84/LPN20	1984	VL		MON-1	46	B			1
AM	Patient 93	MHOM/FR/85/LPN24	1985	VL		MON-1	46	B			1
AM		MCAN/FR/87/LPN37	1987	CanL		MON-1	46	B			1
AM	Patient 94	MHOM/FR/88/LPN46	1988	VL		MON-1	46	B			1
AM	Patient 95	MHOM/FR/89/LPN59	1989	VL	HIV	MON-1	46	B			1
AM	Patient 96	MHOM/FR/93/LPN91	1993	IVL		MON-1	46	B			1
AM	Patient 97	MHOM/00/93/LPN94	1993	VL	HIV	MON-1	46	B			1
AM	Patient 98	MHOM/FR/95/LPN107	1994	IVL		MON-1	46	B			1
AM	Patient 99	MHOM/FR/95/LPN114	1994	IVL		MON-1	46	B			1
AM	Patient 100	MHOM/FR/95/LPN118	1995	VL	HIV	MON-1	46	B			1
AM	Patient 101	MHOM/FR/96/LPN125	1996	VL		MON-1	46	B			1
AM	Patient 102	MHOM/FR/97/LPN148	1997	VL	HIV	MON-1	46	B			1
P	Patient 105	MHOM/FR/97/LPM139	1995	VL	HIV	MON-1	46	B			1
AM	Patient 103	MHOM/FR/97/LPN152	1997	VL		MON-1	46	B			1
AM	Patient 104	MHOM/FR/2001/LPN180	2001	IVL		MON-1	46	B			1
AM	Patient 106	MHOM/FR/2005/LPN257	2005	IVL		MON-1	47	B			1
AM	Patient 107	MHOM/FR/84/LPN21	1984	VL		MON-1	48	B			1
AM	Patient 108	MHOM/FR/92/LPN85	1992	IVL		MON-1	48	B			1
AM	Patient 109	MHOM/FR/2004/LPN240	2004	VL		MON-1	48	B			1
AM	Patient 110	n. d.	2011	IVL		MON-1	49	B			1
AM	Patient 111	MHOM/FR/92/LPN86	1992	IVL		MON-1	50	B			2
AM	Patient 112	MHOM/FR/2001/LPN191	2001	VL	HIV	MON-1	50	B			2
AM	Patient 113	MHOM/FR/2008/LPN321	2008	VL		MON-1	50	B			2
AM	Patient 114	MHOM/FR/99/LPN170	1999	VL		MON-1	51	B			2
AM	Patient 115	MHOM/FR/2006/LPN281	2006	VL		MON-1	52	B			2
CE		MCAN/FR/2006/LPN285	2006	CanL		MON-1	52	B			2
AM	Patient 116	MHOM/FR/87/LPN36	1987	VL	HIV	MON-1	53	B			2
AM	Patient 117	MHOM/FR/91/LPN71	1991	VL	HIV	MON-1	53	B			2
AM	Patient 118	MHOM/FR/93/LPN96	1992	VL		MON-1	53	B			2
AM	Patient 119	MHOM/FR/93/LPN99	1993	VL	HIV	MON-1	53	B			2
AM		MCAN/FR/94/LPN102	1994	CanL		MON-1	53	B			2
AM	Patient 120	MHOM/FR/95/LPN117	1995	VL		MON-1	53	B			2
AM	Patient 121	MHOM/FR/2001/LPN189	2001	VL		MON-1	53	B			2
AM	Patient 122	MHOM/FR/78/LPN1	1978	VL		MON-1	54	B			2
AM		MCAN/FR/82/LPN6	1982	CanL		MON-1	55	B			2
AM	Patient 123	MHOM/FR/84/LPN22	1983	VL		MON-1	55	B			2
AM	Patient 124	MHOM/FR/86/LPN31	1986	VL		MON-1	55	B			2
AM	Patient 125	MHOM/FR/88/LPN48	1988	CL		MON-1	55	B			2
AM	Patient 126	MHOM/FR/89/LPN55	1989	IVL		MON-1	55	B			2
AM	Patient 127	MHOM/FR/90/LPN68	1990	IVL		MON-1	55	B			2
AM	Patient 128	MHOM/FR/92/LPN76	1992	VL		MON-1	55	B			2
AM	Patient 129	MHOM/FR/92/LPN77	1992	VL	HIV	MON-1	55	B			2
AM	Patient 130	MHOM/FR/92/LPN80	1992	VL	HIV	MON-1	55	B			2
AM	Patient 131	MHOM/FR/92/LPN82	1992	VL	HIV	MON-1	55	B			2
CO	Patient 146	MHOM/FR/93/LPN95	1993	VL		MON-1	55	B			2
AM	Patient 132	MHOM/FR/94/LPN106	1994	VL		MON-1	55	B			2
AM	Patient 133	MHOM/FR/94/LPN109	1994	VL	HIV	MON-1	55	B			2
AM	Patient 135	MHOM/FR/96/LPN133	1996	IVL		MON-1	55	B			2
AM	Patient 134	MHOM/FR/96/LPN130	1996	VL		MON-1	55	B			2
AM		MFEL/FR/96/LPN139	1996	CatL		MON-1	55	B			2
AM	Patient 136	MHOM/FR/97/LPN155	1997	VL		MON-1	55	B			2
AM	Patient 137	MHOM/FR/97/LPN158	1997	VL		MON-1	55	B			2
AM	Patient 139	MHOM/FR/2000/LPN176	2000	VL		MON-1	55	B			2
AM	Patient 138	MHOM/FR/2000/LPN175	2000	VL		MON-1	55	B			2
AM	Patient 140	MHOM/FR/2003/LPN209	2003	IVL		MON-1	55	B			2
AM	Patient 141	MHOM/FR/2003/LPN217	2003	VL		MON-1	55	B			2
AM	Patient 142	MHOM/FR/2006/LPN259	2006	VL		MON-1	55	B			2
AM	Patient 143	MHOM/FR/2006/LPN282	2006	IVL		MON-1	55	B			2
AM	Patient 144	MHOM/FR/2007/LPN316	2007	VL		MON-1	55	B			2
AM	Patient 145	MHOM/FR/2011/LPN357	2011	VL		MON-1	55	B			2
AM	Patient 147	MHOM/FR/96/LPN127	1996	VL	HIV	MON-1	56	B			2
AM		MCAN/FR/82/LPN10	1982	CanL		MON-1	57	B			2
AM	Patient 148	MHOM/FR/89/LPN53	1989	VL		MON-1	57	B			2
AM	Patient 149	MHOM/FR/90/LPN66	1990	VL		MON-1	57	B			2
AM	Patient 150	MHOM/FR/93/LPN90	1993	VL		MON-1	57	B			2
AM	Patient 151	MHOM/FR/93/LPN93	1993	IVL		MON-1	57	B			2
AM	Patient 152	MHOM/FR/93/LPN98	1993	VL	HIV	MON-1	57	B			2
AM	Patient 153	MHOM/FR/94/LPN110	1994	VL	HIV	MON-1	57	B			2
AM	Patient 154	MHOM/FR/96/LPN140	1996	IVL		MON-1	57	B			2
AM	Patient 155	MHOM/FR/97/LPN156	1997	VL	HIV	MON-1	57	B			2
AM	Patient 156	MHOM/FR/2000/LPN178	2000	IVL		MON-1	57	B			2
AM	Patient 157	MHOM/FR/2001/LPN190	2001	IVL		MON-1	57	B			2
AM	Patient 158	MHOM/FR/2006/LPN277	2006	VL		MON-1	57	B			2
AM	Patient 159	MHOM/FR/2011/LPN351	2011	IVL		MON-1	57	B			2
AM	Patient 160	MHOM/FR/2011/LPN356	2011	IVL		MON-1	57	B			2
AM	Patient 161	MHOM/FR/2011/LPN366	2011	IVL		MON-1	57	B			2
AM	Patient 162	MHOM/FR/2004/LPN237	2004	VL		MON-1	58	B			2
CO	Patient 163	MHOM/FR/94/LPN105	1994	VL	HIV	MON-1	59	B			1
P	Patient 164	MHOM/FR/96/LPM154	1996	IVL		MON-1	60	A	3/4/5/2	1	
P	Patient 165	MHOM/FR/97/LPM174	1997	VL	HIV	MON-1	61	A	4/3/2	1	
AM	Patient 166	MHOM/FR/99/LPN173	1999	IVL		MON-1	62	A/B			
P	Patient 167	MHOM/FR/97/LPM170	1997	IVL		MON-1	63	A	1	4	
P	Patient 168	MHOM/FR/97/LPM177	1997	VL	HIV	MON-1	63	A	1	4	
P	Patient 169	MHOM/FR/2000/LPM201	2000	VL	HIV	MON-1	63	A	1	4	
P	Patient 170	MHOM/FR/2001/LPM209	2001	VL	HIV	MON-1	64	A	1/3/4/2	4/1/2	
P	Patient 171	MHOM/FR/97/LPM166	1997	VL	HIV	MON-1	65	A	1	4	
P	Patient 172	MHOM/FR/2004/LPM236	2004	IVL		MON-1	66	A	1	4	
P	Patient 173	MHOM/FR/2006/LPM252	2006	VL		MON-1	67	A	1	4	
P	Patient 174	MHOM/FR/94/LPM116	1994	VL	HIV	MON-1	68	A	1	4	
P	Patient 175	MHOM/FR/96/LPM138	1996	VL	HIV	MON-1	68	A	1	4	
P	Patient 176	MHOM/FR/99/LPM189	1999	IVL		MON-1	68	A	1	4	
P	Patient 177	MHOM/FR/2000/LPM204	2000	IVL		MON-1	68	A	1	4	
P	Patient 178	MHOM/FR/98/LPM183	1998	VL	HIV	MON-1	69	A	1/3/2	4	
P	Patient 179	MHOM/FR/97/LPM178	1997	UK		MON-1	69	A	1/3/2	4	
P	Patient 180	MHOM/FR/2003/LPM232	2003	IVL		MON-1	70	A	1	4	
P	Patient 181	MHOM/FR/96/LPM152	1996	VL		MON-1	71	A	1/3/2	4	
P	Patient 182	MHOM/FR/97/LPM168	1997	VL	HIV	MON-1	72	A	1	4	
P	Patient 183	MHOM/FR/98/LPM181	1998	MCL		MON-1	73	A	5	2	
P	Patient 184	MHOM/FR/96/LPM156	1996	IVL		MON-1	74	A	5/1/4/2	2/4/1	
CE		IPER/FR/84/LEM576*	1984	PHLE		MON-1	75	A	4/1/2	1/4	
CE		IARI/FR/84/LEM595*	1984	PHLE		MON-1	75	A	4/1/2	1/4	
AM	Patient 185	MHOM/FR/96/LPN129	1996	VL	HIV	MON-1	76	A	1	4	
AM	Patient 186	MHOM/FR/88/LPN45	1988	VL	HIV	MON-1	77	A	1	4	
AM	Patient 187	MHOM/FR/89/LPN58	1989	IVL		MON-1	77	A	1	4	
AM	Patient 188	MHOM/FR/91/LPN69	1991	VL	HIV	MON-1	77	A	1	4	
AM	Patient 189	MHOM/FR/96/LPN128	1996	VL	HIV	MON-1	77	A	1	4	
AM	Patient 190	MHOM/FR/97/LPN151	1996	VL	HIV	MON-1	77	A	1	4	
AM	Patient 86	MHOM/FR/97/LPN153	1997	VL	HIV	MON-1	77	A	1	4	
AM		MCAN/FR/98/LPN168	1998	CanL			77	A	1	4	
AM	Patient 86	MHOM/FR/2000/LPN177	2000	RVL	HIV	MON-1	77	A	1	4	
AM	Patient 86	MHOM/FR/2001/LPN185	2001	RVL	HIV	MON-1	77	A	1	4	
AM	Patient 191	MHOM/FR/2001/LPN186	2001	VL	HIV	MON-1	77	A	1	4	
AM	Patient 86	MHOM/FR/2003/LPN215	2003	RVL	HIV	MON-1	77	A	1	4	
AM	Patient 192	MHOM/FR/2007/LPN314*	2007	VL	HIV	MON-1	78	A	1/6	4/3/1	
AM		MCAN/FR/2006/LPN267	2006	CanL		MON-1	79	A	1/6/3/4	4/2/3	
P	Patient 193	MHOM/FR/2002/LPM222	2002	VL	HIV	MON-1	80	A	1/5/6	4/2	
P	Patient 194	MHOM/FR/2001/LPM214	2001	VL		MON-1	81	A	2/4	1	
P	Patient 195	MHOM/FR/2002/LPM226	2002	VL	HIV	MON-1	82	A	2/4	1	
P	Patient 196	MHOM/FR/2002/LPM228	2002	VL		MON-1	83	A	2/4	1	
P	Patient 197	MHOM/FR/96/LPM155	1996	VL		MON-1	84	A	2/3/1	1	
P	Patient 198	MHOM/FR/96/LPM159	1996	VL		MON-1	84	A	2/3/1	1	
P	Patient 199	MHOM/FR/94/LPM133	1994	VL	HIV	MON-1	85	A	2	1	
P	Patient 200	MHOM/FR/96/LPM161	1996	IVL	HIV	MON-1	85	A	2	1	
P	Patient 201	MHOM/FR/2009/LPM264	2009	MCL		MON-1	85	A	2	1	
P	Patient 202	MHOM/FR/94/LPM122-2	1994	VL	HIV	MON-1	86	A	1/3/4	4/1	
P	Patient 203	MHOM/FR/2001/LPM212	2001	CL		MON-1	87	A	4/3/2	1	
P	Patient 204	MHOM/FR/2008/LPM260	2008	VL	HIV	MON-1	88	A	2	1/3	
P	Patient 205	MHOM/FR/99/LPM191	1999	CL	HIV	MON-1	89	A	1/3	1/4	
P	Patient 206	MHOM/FR/95/LPM137	1995	VL	HIV	MON-1	90	A	3/6	1/3	
P	Patient 207	MHOM/FR/2000/LPM196	2000	VL	HIV	MON-1	91	A	4	1	
P	Patient 208	MHOM/FR/94/LPM118	1994	VL	HIV	MON-108	92	A	4/1/5	1/2/4	
CE	Patient 212	MHOM/FR/85/LEM716*	1985	VL		MON-1	93	A	4	1	
P	Patient 210	MHOM/FR/97/LPM176	1997	IVL		MON-1	93	A	4	1	
CO	Patient 209	MHOM/FR/98/LPM186	1998	VL		MON-1	93	A	4	1	
P	Patient 211	MHOM/FR/2001/LPN183	2001	VL		MON-1	93	A	4	1	
AM	Patient 213	MHOM/FR/83/LPN18	1983	VL		MON-1	94	A	4	1	
CE	Patient 214	MHOM/FR/87/LEM1098*	1987	CL		MON-1	94	A	4	1	
CE	Patient 215	MHOM/FR/78/LEM75*	1978	VL		MON-1	94	A	4	1	
CE	Patient 216	MHOM/FR/85/LEM663*	1985	VL		MON-1	95	A	4	1	
AM	Patient 217	MHOM/FR/88/LPN41	1988	VL	HIV	MON-1	96	A	2/5	3/2	
AM	Patient 218	MHOM/FR/2003/LPN213	2003	VL		MON-1	97	A	4	1	
AM	Patient 219	MHOM/FR/96/LPM162	1996	CL		MON-24	98	A	6	3	
AM	Patient 220	MHOM/00/2004/LPN234	2004	CL		MON-24	99	A	6	3	
AM	Patient 190	MHOM/FR/2001/LPN182	2001	RVL	HIV	MON-1	100	A	1	4	
AM	Patient 190	MHOM/FR/2001/LPN188	2001	RVL	HIV	MON-1	100	A	1	4	
P	Patient 221	MHOM/FR/2000/LPM200	2000	VL	HIV	MON-1	101	A	2/1	1/3/4	
P	Patient 222	MHOM/FR/94/LPM120	1994	VL	HIV	MON-1	102	A	2/1/3	1/4	
P	Patient 223	MHOM/FR/99/LPM194	1999	MCL		MON-1	103	A	4/3/2	1	
P	Patient 224	MHOM/FR/96/LPM158	1996	VL		MON-1	104	A	4/2/5	1/2	
AM		MCAN/FR/80/LPN4	1980	CanL		MON-1	105	A	4/3/2	1	
AM		MCAN/FR/82/LPN8	1982	CanL		MON-1	105	A	4/3/2	1	
AM	Patient 225	MHOM/FR/99/LPN171	1999	IVL		MON-1	106	A	3	1	
P	Patient 226	MHOM/FR/2001/LPM211	2001	VL	HIV	MON-1	107	A	3	1	
P	Patient 227	MHOM/00/00/LPM148	1995	UK		MON-1	108	A	3	1	
AM	Patient 228	n. d.	2011	VL		MON-1	109	A	3	1	
AM		MCAN/FR/86/LPN27	1986	CanL		MON-1	110	A	3	1	
AM	Patient 229	MHOM/FR/99/LPN174	1999	CL		MON-24	111	A	6	3	
AM	Patient 230	MHOM/FR/2005/LPN253	2001	VL	HIV	MON-80	112	A	6	3	
P	Patient 231	MHOM/00/2003/LPM233	2003	IVL		MON-1	113	A	2	3	
CO	Patient 232	MHOM/FR/2000/LPM205	2000	VL		MON-1	114	A	6	3	
CO	Patient 233	MHOM/FR/92/LPN83	1991	CL	HIV	MON-1	115	A	6	3	
CO	Patient 234	MHOM/FR/99/LPM190	1999	VL	HIV	MON-1	116	A	6	3	
AM	Patient 235	MHOM/FR/89/LPN60	1989	VL	HIV	MON-1	117	A	2	1/3	
P	Patient 236	MHOM/FR/2008/LPM259	2008	VL	HIV	MON-1	118	A	2/1	1/4/3	
P	Patient 237	MHOM/FR/2007/LPM254	2007	IVL		MON-1	119	A	6	3	
AM	Patient 238	MHOM/FR/93/LPN97	1993	CL		MON-24	120	A	6	3	
P	Patient 239	MHOM/FR/95/LPM136	1995	VL	HIV	MON-1	121	A	6	3	

Legend of columns: Endemic area: AM: Alpes-Maritimes; P: Provence; CE: Cévennes; CO: Corsica. Patients: Anonymized name given to the patients. Sample WHO code: WHO code of the isolates; The MLMT profile of the samples indicated with * was already characterized by Hide et al [9]; n. d. = not defined. Y: Year of isolation. C F / H: Clinical form of the disease and/or host: VL–visceral leishmaniasis; IVL–Infant under 15 years with visceral leishmaniasis; CL–cutaneous leishmaniasis; MCL–muco-cutaneous leishmaniasis; RVL—New episode of leishmaniasis in VL patient; AC–asymptomatic carrier; CanL–canine leishmaniasis; CatL–leishmaniasis in cat; PHLE–sample isolated from phlebotomine sandfly; UK–unknown. Zymo: zymodeme of isolate. G: genotypes. P: population as defined by STRUCTURE.

**Fig 1:**
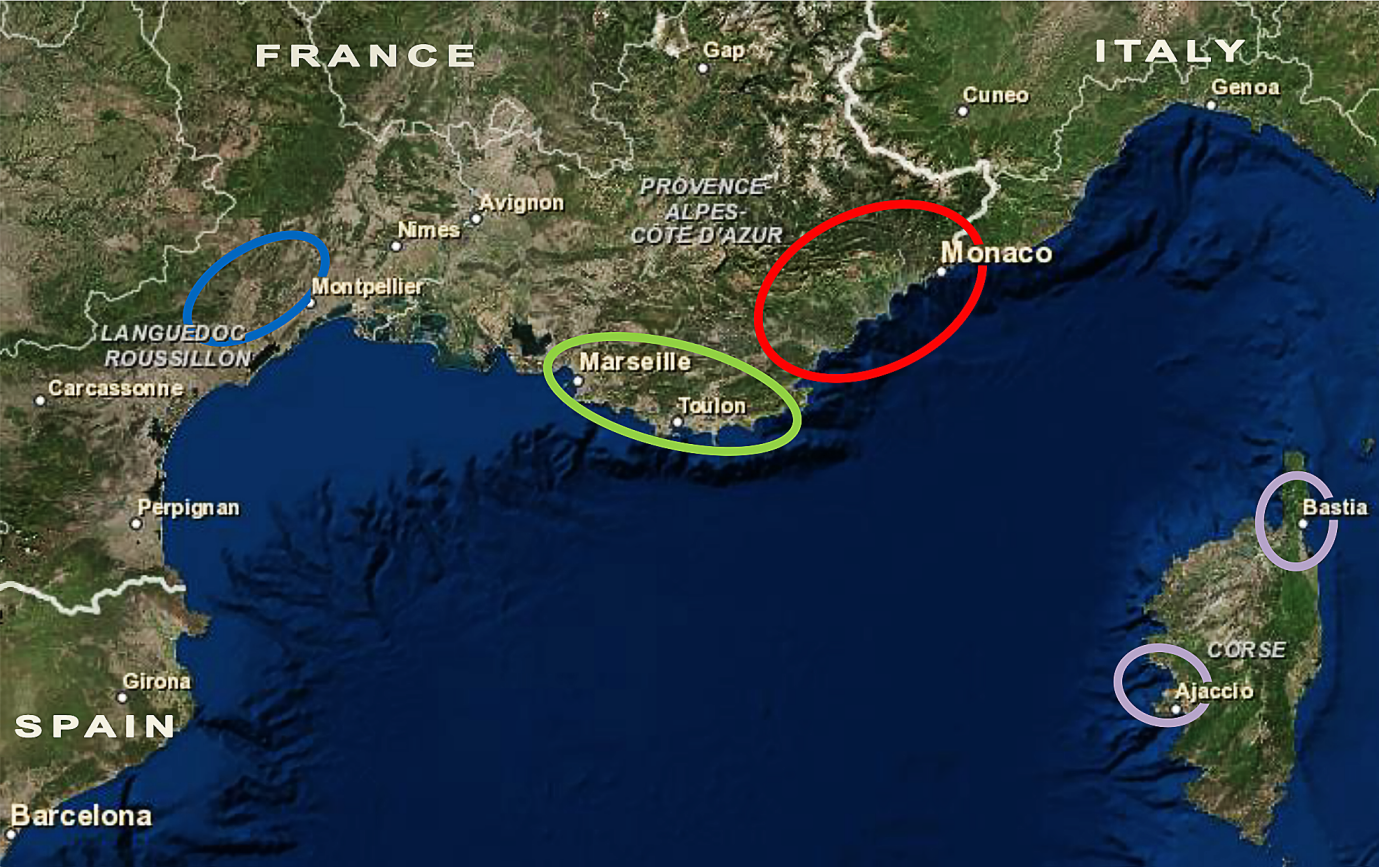
Geographical distribution of isolates from Southeastern France. Isolates clustered into four endemic areas. The geographic areas where samples were isolated from Cévennes (blue), Provence (green), Alpes-Maritimes (red) and Corsica (violet) are indicated. The Alpes-Maritimes and Provence endemic areas are located in the PACA region.

Concerning the geographical distribution (Fig 1), the samples were collected from the endemic areas of AM (n = 178), P (n = 75), CO (n = 9) and CE (n = 8). The samples were isolated at the University Hospitals of Nice, Marseille and Montpellier (France) between 1978 and 2011. MLEE typing and cryoconservation were performed at the Centre National de Référence des *Leishmania* (*Leishmania* collection, BRC-Leish, Montpellier, France, BioBank N° BB-0033-00052). Overall, 259 isolates were characterized as zymodeme MON-1, six were MON-24, one was MON-11, one was MON-80 and one was MON-108. The data were unavailable for two isolates. For this study, the primary cultures from the patient stored at -80°C were thawed and the cells were cultured for six days before harvesting for DNA extraction.

### DNA extraction

The microsatellite data indicated with an asterisk in Table 1 were obtained from a previous study [9].

DNA of the remaining isolates was extracted from promastigotes grown in Schneider’s insect medium (Sigma Aldrich, France) supplemented with serum calf, urine, penicillin, streptomycin and L-glutamine (Sigma Aldrich). Promastigotes were harvested on the sixth day of culture, and DNA was extracted from a pellet of 2X10^8^ parasites using a QIAamp DNA mini kit (Qiagen, France) according to the manufacturer’s instructions. DNA extracted from the strain MHOM/FR/85/LEM716 was used as a control to determine the size of amplified microsatellite fragments, as this microsatellite data have been published [9].

### Multi-Locus Microsatellite Typing (MLMT)

Twelve microsatellite loci were amplified using the PCR conditions as previously described: LiBTG, LiBTA, LIST7021, LIST7025, LIST7026, LIST7031, LIST7033, Li22-25, Li45-24, TubCA, Li71-5/2 and Rossi2 [9–12]. The amplification products were analyzed using an automated capillary ABI Prism 3130XL Genetic Analyzer (Applied Biosystems, France). The data were stored and analyzed using GeneMapper analysis software (version 4.0, Applied Biosystems). PCR fragment sizes were determined using the internal size standard GeneScan 500 LIZ (Applied Biosystems). All 270 *Leishmania* isolates were genotyped at each of the 12 loci. With the PCR fragment size of the control strain MHOM/FR/85/LEM716, we were able to include microsatellite data from the Hide et al. study (data indicated with an asterisk in Table 1). Four isolates from this previous study (MCAN/FR/95/LPN122, MCAN/FR/95/LPN123, MCAN/FR/95/LPN124, and MCAN/FR/95/LPN124) were re-extracted from culture and re-genotyped blindly. The same microsatellite results described by Hide et al. were obtained [8].

### Genetic diversity and differentiation analysis

Descriptive statistics for the observed genetic populations were calculated using Genetix version 4.05.2 (2004) and FSTAT Version 2.9.3.2 [13]. Using these programs, we calculated allelic diversity (number of allelic variants per maker), expected (*He*) and observed (*Ho*) heterozygosity, genetic diversity within subsamples *Hs*, inbreeding coefficient (*F*_IS_) the migration rates (gene flow) (*Nm*) [14]. The *Fst* value, which indicates the degree of genetic differentiation and gene flow among populations was also calculated. *Fst* values above 0.25 with significant *p*- values (<0.05) indicated strong genetic differentiation [15].

### Clustering and phenetic analyses

Phylogenetic analyses were performed based on the microsatellite profiles. A distance matrix was calculated using the Chord distance (Cavalli-Sforza and Edwards 1967) setting in the POPULATIONS 1.2.31 software with bootstrap values determined for 1,000 replicates (http://bioinformatics.org/~tryphon/populations/) [16]. The resulting distance matrix was processed using MEGA 4.0.2 to construct an unrooted Neighbor-Joining (NJ) tree [17].

The genetic characteristics of the *Leishmania* samples were also investigated using a model-based Bayesian clustering method implemented in STRUCTURE v 2.3.4 [18]. This algorithm simultaneously estimates the allele frequencies to assign individuals into genetically distinct populations (*K*) and each probability for the identification of the most likely number of populations. A series of ten independent runs was performed for each K value between one and ten. The following parameters were used: burn in period of 20,000 iterations, 200,000 Markov Chain Monte Carlo iterations and admixture model. The most probable number of clusters was identified via calculation of the Delta *K* (*ΔK*), which is based on the rate of change in the log probability of data between successive K values. The peak of the *ΔK* graph corresponds to the most probable number of populations in the data set [19].

### Statistical analysis

A chi-square statistical test was performed to determine whether the observed data differed significantly from the expected ratios. The chi-square value was considered significant when p≤0.05. This test was used to compare the proportion of HIV+ patients in Populations A and B.

## Results

### Microsatellite analysis

In total, 270 isolates were typed at 12 microsatellite markers with one or two alleles at each locus. All markers were polymorphic. The number of alleles per locus (*N*a) ranged from 3 to 13. Li22-35, LIST7021, LiBTG, LiBTA and LIST7026 were the most polymorphic markers with 13, 12, 10, 9 and 8 alleles, respectively. LIST7031, Li71-5/2 and LIST7025 were the least polymorphic markers with three different alleles (Table 2).

**Table 2:** Descriptive statistics of the 270 isolates analyzed at the 12 microsatellite markers.

Locus	Na	*He*	*Ho*	*Hs*	*F*_IS_
Li22-35	13	0.644	0.082	0.645	0.874
LIST7021	12	0.547	0.037	0.548	0.933
LiBTG	10	0.620	0.041	0.621	0.935
LiBTA	9	0.809	0.174	0.811	0.786
LIST7026	8	0.690	0.033	0.691	0.952
TubCA	7	0.541	0.041	0.542	0.925
Li45-24	6	0.649	0.026	0.650	0.960
Rossi2	6	0.553	0.019	0.554	0.967
LIST7033	6	0.533	0.019	0.534	0.965
LIST7031	3	0.230	0.026	0.231	0.888
LIST7025	3	0.240	0.019	0.224	0.918
Li71-5/2	3	0.502	0.011	0.503	0.978
Overall	7.17	0.545	0.044	0.546	0.920

*Na*: number of alleles per locus; *He*: expected heterozygosity; *Ho*: observed heterozygosity; *Hs*: genetic diversity; *F*_IS_: inbreeding coefficient.

Genetic variability was analyzed among the 12 microsatellite loci (Table 2). The *Ho* was weak and ranged from 0.011 to 0.174 for Li71-5/2 and LiBTA, respectively, with an overall *Ho* at 0.044. The mean intra-population *Hs* was 0.546 (0.224–0.811) for the entire sample set and 0.531 (0.208–0.804) for the MON-1 population (Table 2). The *F*_IS_ for the entire population was 0.920, thereby indicating a considerable degree of inbreeding. A separate analysis was performed to investigate the genetic polymorphisms among the four geographically determined populations (Table 3). Extensive inbreeding in the four populations was observed, with the highest inbreeding coefficient found in the populations of the CO and AM endemic areas. The genetic differentiation among the four endemic areas was tested using FSTAT version 2.9.3.2 (Table 4). The *F*st values ranged from 0.067 to 0.321. All *F*st values between the four endemic areas were significant. We obtained lower values for P versus CE and P versus CO and higher values for AM versus CE and CO versus CE (which may be due to the low number of isolates collected from CE and CO). When comparing AM and P samples from 1993 to 2009 (corresponding to the time period of the isolation of samples from the P endemic area used in this study), the *F*st value obtained was similar to the *F*st value corresponding to the complete period of sample collection (1978 to 2011).

**Table 3:** Genetic diversity among the four endemic areas based on the MLMT profiles of the 12 analyzed markers.

	Na	*He*	*Ho*	*F*_IS_
AM	6.333	0.462	0.030	0.936
P	5.417	0.498	0.079	0.844
CO	3.000	0.568	0.028	0.956
CE	2.250	0.281	0.042	0.869

*Na*: Number of alleles per locus, *He*: expected heterozygosity, *Ho*: observed heterozygosity, *F*_IS_: inbreeding coefficient.

AM = Alpes-Maritimes, P = Provence, CO = Corsica and CE = Cévennes.

**Table 4:** Genetic differentiation between the isolates from the four endemic areas analyzed using *F*-statistics with the corresponding *p*-values.

Endemic areas	Number of isolates	*F*st	p-value
**P vs. AM**	**75 / 178**	**0.239**	**0.008**
**P vs. CO**	**75 / 9**	**0.126**	**0.008**
**P vs. CE**	**75 / 8**	**0.067**	**0.050**
**CO vs. CE**	**9 / 8**	**0.270**	**0.008**
**AM vs. CO**	**178 / 9**	**0.161**	**0.008**
**AM vs. CE**	178 / 8	**0.321**	**0.008**
**P vs. AM isolates collected during same period of time (1993 to 2009)**	**75 / 111**	**0.236**	**0.050**
**Sub-population defined according to their position in relation to the Vars river**			
**AM East Vars vs. AM West Vars**	**122 / 50**	**0.062**	**0.050**
**AM West Vars vs. P**	**52 / 75**	**0.144**	**0.050**
**P vs. AM East Vars**	**75 / 122**	**0.308**	**0.050**
**Sub-population within the P endemic area**			
Within P: Marseille vs. Toulon	42 / 7	0.033	0.850
Within P: Marseille vs. Other cities In P	42 / 33	0.012	0.550

Sub-populations were also defined according to their position in relation to Vars River (S1 Table). This river is located in the southeast of France and flows in the Alpes-Maritimes Department. The *F*st values highlighted a gradient of differentiation from the East Vars to the West Vars and up to the P endemic area. A high and significant genetic differentiation (*F*st = 0.308) was obtained when comparing isolates from east of the Vars River and the P endemic area. The comparison of sub-populations isolated from the P endemic area failed to show any genetic differentiation (Marseille versus Toulon or Marseille versus other cities in the P endemic area), with the limitation that few samples were collected from Toulon (n = 7).

### Geographical distribution of isolates from the two main endemic areas of AM and P

**Fig 2:**
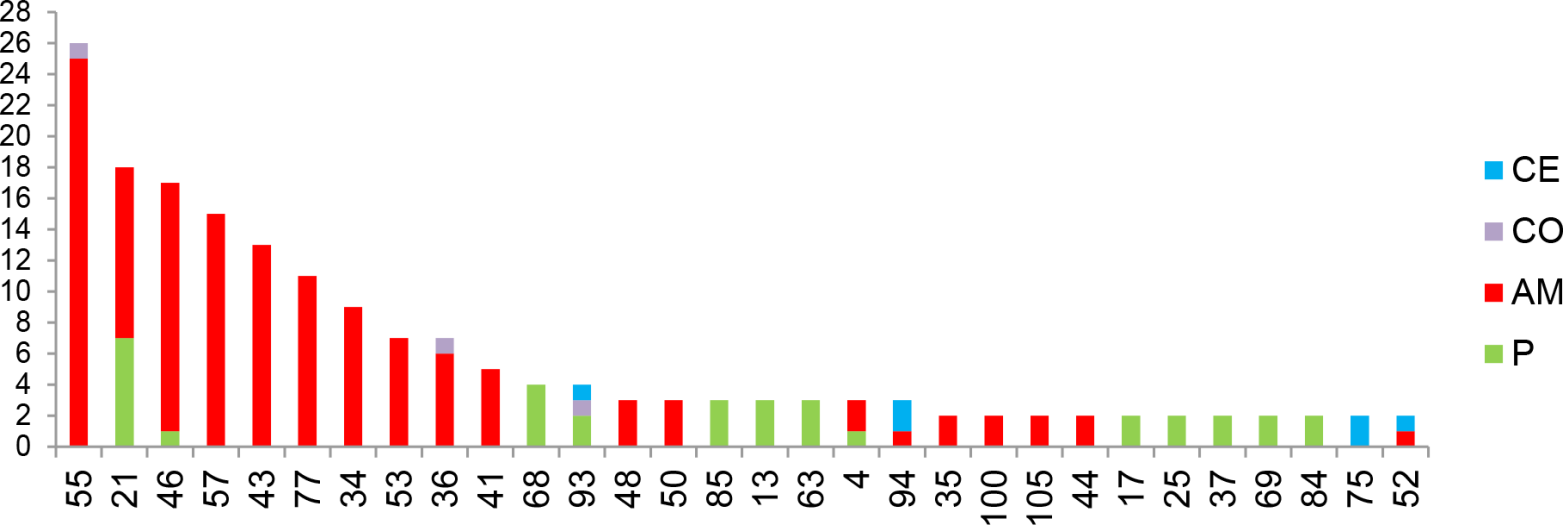
Repeated genotypes. Arbitrary numbers assigned to the 30 repeated genotypes in (x-axis) and number of isolates belonging to each genotype (y-axis). The origin of each isolates is indicated by the different colors.

**Fig 3:**
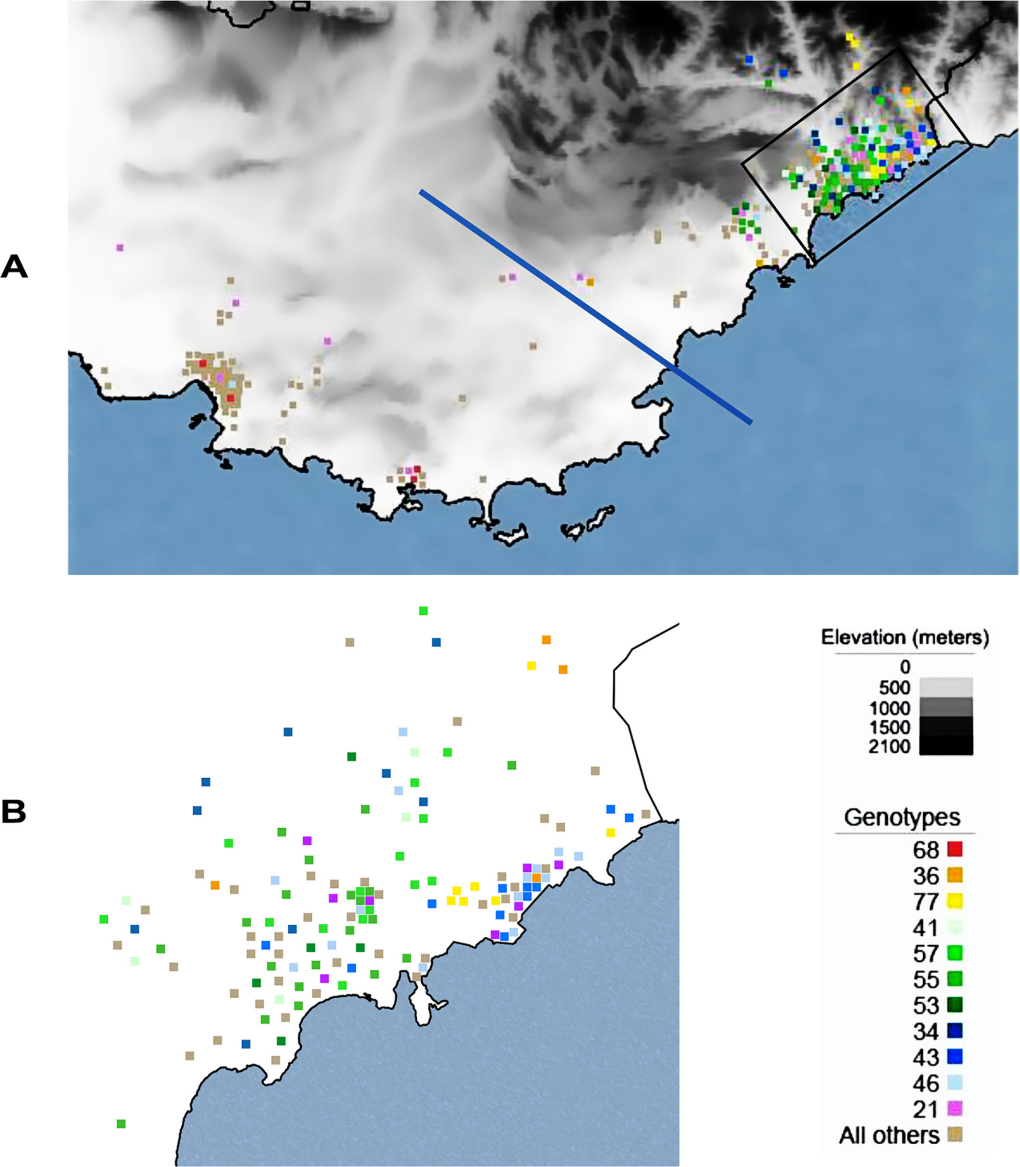
A. Localization of the genotypes. Eleven repeated genotypes, which were observed in four or more isolates, are represented with colored squares on the map of the endemic areas of Alpes-Maritimes and Provence. The two endemic areas are separated by a blue line. The remaining genotypes are represented by light brown squares. The area in the black rectangle is shown in further detail in Fig 3B. B. Detailed localization of isolates in the Alpes-Maritimes endemic area and more precisely the surrounding region of Nice. Isolates were localized at the city level. The black line represents the delimitation of Nice.

A total of 121 different genotypes were identified from the 270 isolates corresponding to a genotype frequency of 0.037. The entire sample set comprised 91 unique genotypes (75%). Among the 30 repeated genotypes, seven were common to two endemic areas, one was common to three endemic areas and 22 pertained to the same focus (Fig 2). The genotypes 21 (primarily) and 46 were found in both endemic areas of AM and P (Fig 2). Within these endemic areas, Faucher et al. have described high- and low-risk sub-areas of VL [5]. In our study, the repeated genotypes were almost exclusively found in high-risk areas, with the only exception of genotype 21 which was found in high and low risk sub-areas (Fig 3A and 3B). Among the 30 repeated genotypes, 11 were found in four or more samples. These 11 genotypes represented 132 isolates.

### Temporal analysis of genotypes

**Fig 4:**
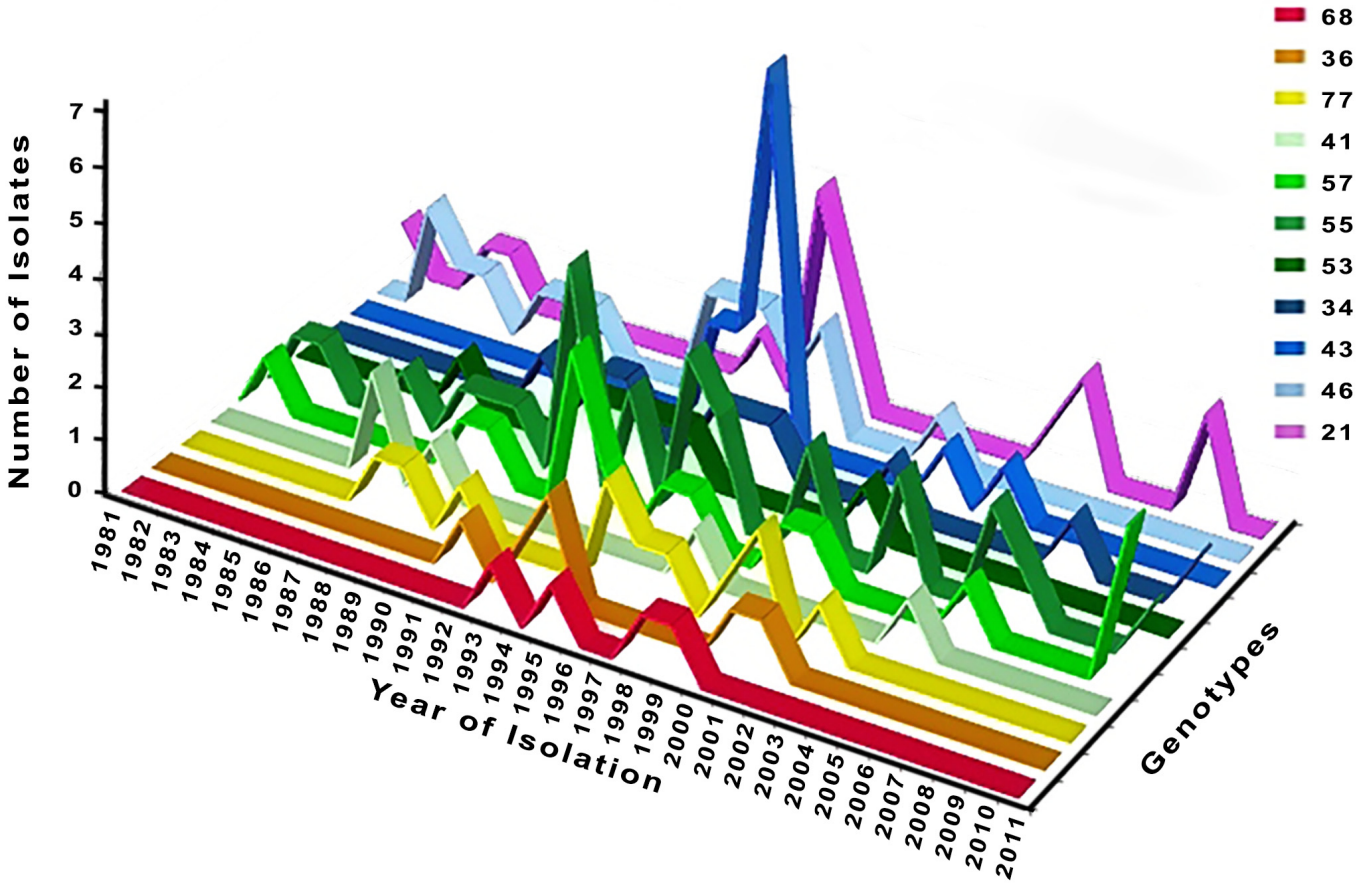
Temporal distribution of the 11 genotypes represented by more than four isolates.

The isolates were collected over the course of a 33-year period from 1978 to 2011 (Fig 4). The repeated genotypes, found in four or more samples, were isolated over a period of six (genotype 68) to 29 years (genotypes 57 and 55). All genotypes except 68, 36, 34 and 43 were isolated from both humans and dogs. Genotype 21 was found to be present in the AM and P endemic areas over the course of 28 years. This genotype was first isolated in 1981 in the AM endemic area and then in 1996, at which time it was first identified in both the AM and P endemic areas. After 1996, genotype 21 was only isolated in the P. Finally, in 2009, this genotype was found in both the AM and P endemic areas. Genotype 21 was isolated from a variety of patients including asymptomatic carriers (AC), adult VL, infant VL and HIV+ VL cases.

### Relapse and re-infection

Four patients (patients 4, 64, 86 and 190) experienced more than one episode of VL (Table 1). Three of these patients were HIV+ (patients 4, 86 and 190). The isolates derived from patient 4 and 64 (HIV+ and HIV-, respectively), which were collected during a first VL episode (2003 for patient 4 and 2001 for patient 64) and second VL episode (one year later), presented identical MLMT profiles. In both cases, relapse was suspected. Patient 190 (HIV+) presented three episodes of VL, for which the second and third (2001 for both episodes) isolates differed from the first isolate (1996) at only one marker (LIST7021). Re-infection was suspected for this patient; however, further studies are warranted to confirm whether only one allelic change can lead to the suspicion of a re-infection or reflects the evolution of the isolate over time. Patient 86 (HIV+) presented five different episodes of VL due to active chronic VL [20]. Isolates from the second (2000), third (2001) and last episode (2003) presented with the same MLMT profile compared with the isolate from the initial infection (1997). However, the MLMT profile of the fourth episode (2002) isolate varied at 11 loci; only Rossi2 remained the same. Re-infection and relapse were suspected for this patient. Each isolate of these multiple episodes was characterized zymodeme MON-1.

### Association between genotype and clinical manifestation

Genetic differentiation among the various populations was tested using FSTAT Version 2.9.3.2.

In France, at the end of the 1990s and early 2000s, the repellent collar had been widely use to protect dogs from parasitic transmission [21,22]. To determine whether this had an impact on genetic differentiation, we compared the isolates before and after the introduction of repellent collar in the AM and P endemic areas. To minimize the temporal effect on genetic variability and determine whether the repellent collar led to a bottleneck effect, the samples collected between 1996 and 2004 were excluded (Table 5). No differentiation was found in both endemic areas, thereby suggesting no bottleneck effect due to repellent collar use.

**Table 5:** Differentiation measures (*Fst*) and testing (p-value) between different *Leishmania infantum* isolates according to the use of collar repellent and clinical manifestations.

Subsamples	Number of isolates	*F*st	p-value
**Effect of collar repellent. Comparison of isolates from 1978 to 1995 and 2005 to 2011 (years 1996–2004 excluded)**			
P endemic area	11 / 12	0.0412	0.15
AM endemic area	87 / 40	≈ 0	0.95
**Comparison of clinical manifestations. Other means VL, CL, MCL**			
AM AC vs other 1991–2001	9 / 172	0.0763	0.10000
AM AC vs HIV isolated from 1994 to 1998 in the same endemic area	11 / 9	0.0830	0.10000
HIV + vs HIV -	95 / 150	0.0683	0.05000
HIV + vs VL adult	95 / 72	0.0857	0.05000
HIV + vs CanL CatL	95 / 21	0.0167	0.50000
P HIV + vs VL adult	40 / 11	0.0020	0.80000
AM HIV + vs VL adult	52 / 50	0.1510	0.05000
IVL vs CanL CatL	58 / 21	-0.0013	0.90000
VL vs IVL	70 / 57	0.0003	0.30000

Nine isolates from asymptomatic carriers were compared with 11 isolates from HIV patients collected between 1994 and 1998 in the same restricted endemic area. To avoid bias, we selected isolates collected two years before and after the date of the collection of the asymptomatic carrier isolates (1996). Unlike the findings reported by Hide et al., no genetic differentiation was observed between isolates from asymptomatic carriers and those derived from HIV+ patients (Table 5) [9]. However, additional isolates from asymptomatic carriers are required to strengthen these findings. A genetic differentiation was observed between isolates from HIV+ and VL adult patients in AM (Table 5).

### Clustering analysis

**Fig 5:**
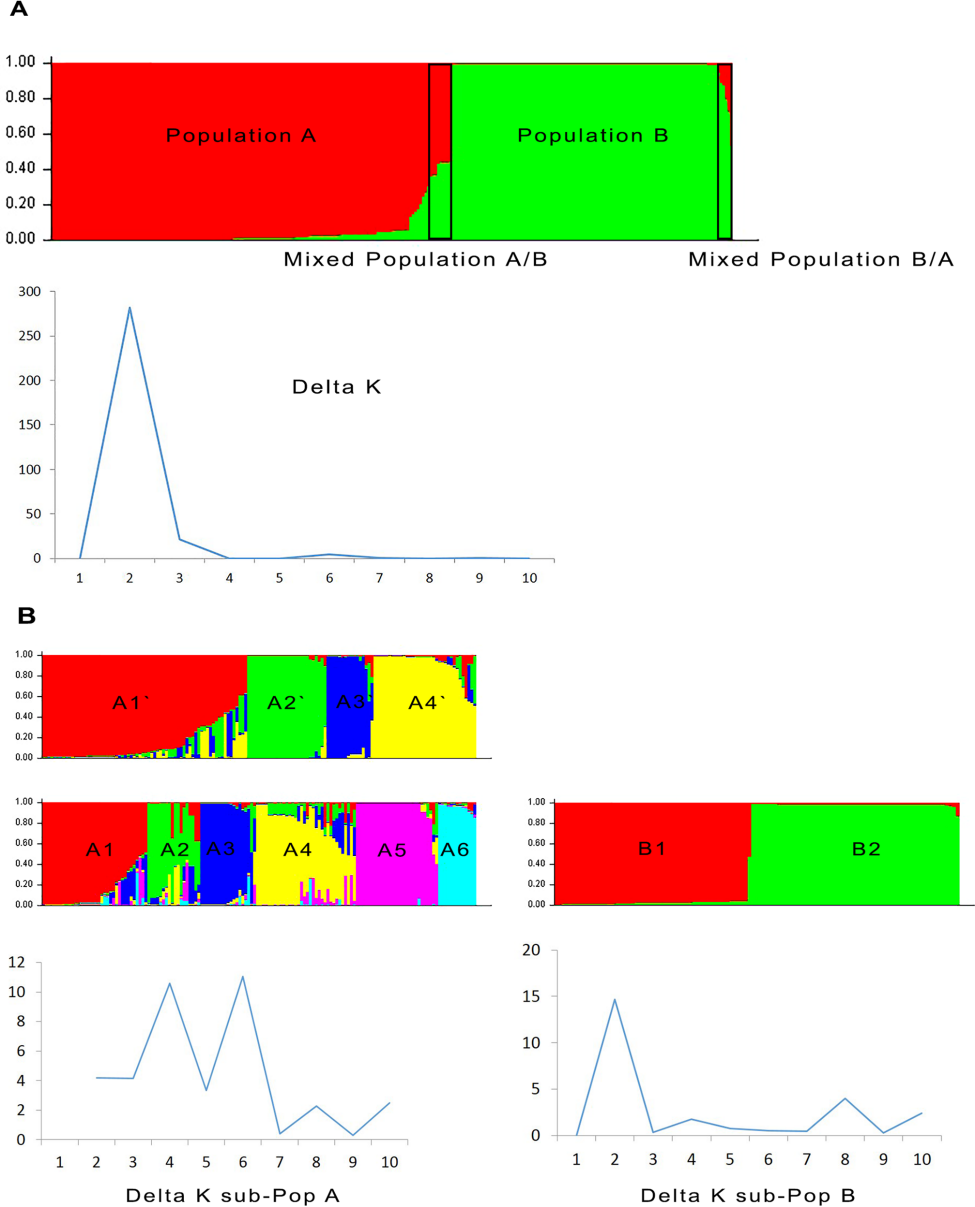
Estimated population structure for *L*. *infantum* from Southeastern France assessed using STRUCTURE software based on the analysis of the 270 *L*. *infantum* DNA samples at 12 microsatellite markers. **A**: The plots show the estimated membership coefficient (Q) of each isolates. Each isolate is represented by a single vertical line divided into K colors, in which K is the number of populations assumed. Each color represents one population, and the length of the colored segment shows the estimated proportion of isolates membership in that population. The derived graph for delta K shows K = 2, thereby indicating the presence of two populations in the investigated sample set. **B**: Isolates of the sub-populations A and B with delta K values for each sub-population. Two close values were observed for K in sub-population A: K = 4 and K = 6. For sub-population B, K = 2.

Bayesian model-based analysis of the 270 isolates using STRUCTURE (with calculation of ΔK) indicated two distinct genetic populations (Fig 5A). Population A consisted of 148 samples: 73 from P, 61 from AM, seven from CO and seven from CE. This population consisted of zymodemes MON-1 and nine isolates with zymodemes other than MON-1. Population B consisted of 109 isolates: 104 from AM, two from P, two from CO and one from CE. Isolates from Population B were all characterized zymodeme MON-1. Among the Populations A and B, 13 isolates had mixed genotypes (11 isolates corresponding to seven genotypes and two isolates corresponding to two genotypes in Populations A and B, respectively). The isolates with mixed genotypes shared allele characteristics of each population. All isolates with mixed genotypes were collected in the AM endemic area and were characterized zymodeme MON-1. Thus, Population A is a mixed population with almost an equivalent number of isolates from AM and P corresponding to MON-1 and all non-MON-1 isolates. The estimated gene flow between isolates from AM and P within Population A (*N*m value) was 8.38. Population A also displayed a marked proportion of isolates from HIV+ patients (44.7%) compared with Population B (21.6%) (p<0.05). The two populations A and B defined by STRUCTURE were significantly different as shown by the *F*st value 0.503 and p-value equal to 0.05. The estimated gene flow between the populations (*N*m value) was 0.25, thereby indicating very few exchanges between those two populations.

The STRUCTURE analysis of Population A separately from Population B, excluding admixed isolates (13 isolates), revealed a delta K graph with two peaks at K = 4 and K = 6 (Fig 5B). The main difference between K = 4 and K = 6 was that sub-population A1’ at K = 4 was split into three populations at K = 6: sub-population A2 (four isolates from sub-population A1’), A3 (16 isolates from sub-population A1’) and A4 (15 isolates from sub-population A1’). Whether at K = 4 or K = 6, the sub-population defined contained isolates from the AM and P endemic areas and from HIV+ patients. The non-MON-1 isolates were grouped into sub-population A1’ and A3’ with K = 4 and into sub-population A2, A4 and A6 with K = 6. No cluster based on clinical data, geographic area or zymodeme profile was found within the defined sub-populations. Some isolates displayed mixed genotypes within the A sub-populations. At K = 4 and K = 6, 26 and 44 isolates had mixed genotypes, respectively. At K = 6, isolates with mixed genotypes came from P (n = 33; 75%), AM (n = 8; 18.2%) and CE (n = 3; 6.8%).

When excluding mixed genotypes, the sub-populations A1, A2, A3, A4, A5 and A6 as well as A1’, A2’, A3’ and A4’ were significantly different, as shown by the significant *F*st values ranging from 0.249 to 0.833 and from 0.322 to 0.813 for K = 4 and K = 6, respectively (Table 6). At K = 4, the highest *Nm* value was obtained between sub-population A1’ and sub-population A3’ (0.53), whereas at K = 6, the highest *Nm* value was 0.75 between sub-population A2 and sub-population A6, thereby indicating only limited genotype flow between these sub-populations (Table 6).

**Table 6:** Differentiation measures, migration rate and statistical significance between different *Leishmania infantum* isolates according to sub-populations defined using STRUCTURE. In sub populations A, the isolates with mixed genotypes have been excluded (26 and 44 isolates for K = 4 and K = 6, respectively). Only the highest and lowest *F*st values are represented in this table.

	Sub populations	Number of Isolates	*F*st	*p*-value	*Nm*
Sub Pop A	Pop A2’ vs A4’	26 / 30	0.813	0.008	0.06
K = 4	Pop A1’ vs A3’	52 / 14	0.322	0.008	0.53
Sub Pop A	Pop A3 vs A5	16 / 26	0.833	0.003	0.05
K = 6	Pop A2 vs A6	7 / 13	0.249	0.003	0.75
Sub Pop B					
K = 2	Pop B1 vs B2	51 / 58	0.537	0.050	0.22

*F*st: degree of genetic differentiation, *Nm*: migration rates, *p*-value: statistical significance.

Two main sub-populations were defined using STRUCTURE for Population B: sub-population B1 and sub-population B2. No mixed genotypes were present. Sub-population B1 consisted of 48 isolates from AM, two from P and one from CO. Sub-population B2 consisted of 56 isolates from AM, one from CO and one from CE. These two sub-populations were genetically different as evidenced by the *F*st value (0.537 and *p*-value = 0.05). Few exchanges occurred between these two sub-populations (*Nm* = 0.22 was obtained between sub-population B1 and sub-population B2).

Sub-populations A3’ (sub-population A K = 4) and A6 (sub-population A K = 6) displayed the highest number of alleles per population (Table 7). The expected heterozygosity (*He*), a measure of genetic diversity, was higher in the A sub-populations with non-MON-1 isolates compared with the A sub-populations with only MON-1 isolates (Table 7). All A sub-populations displayed a high inbreeding coefficient (*F*_IS_) (> 0.7), whereas the sub-population B2 displayed a low inbreeding coefficient (0.343) (Table 7).

**Table 7:** Descriptive statistics per population.

	Name of the sub population	n	*Na*	*He*	*Ho*	*F*_IS_
Sub Pop A	Pop A1’	52	3.500	0.380	0.066	0.830
K = 4	Pop A2’	26	1.500	0.052	0.013	0.763
	Pop A3’	14	5.417	0.695	0.048	0.936
	Pop A4’	30	2.000	0.161	0.014	0.917
Sub Pop A	Pop A1	27	1.917	0.149	0.012	0.920
K = 6	Pop A2	7	2.417	0.395	0.071	0.843
	Pop A3	16	1.917	0.169	0.016	0.913
	Pop A4	15	1.917	0.184	0.006	0.972
	Pop A5	26	1.500	0.052	0.013	0.763
	Pop A6	13	5.000	0.680	0.051	0.931
Sub Pop B	Pop B1	51	1.500	0.080	0.011	0.860
K = 2	Pop B2	58	1.917	0.067	0.0445	0.34337

In sub populations A, the isolates with mixed genotypes have been excluded (26 and 44 isolates for K = 4 and K = 6, respectively). n: number of isolates per population; *Na*: number of alleles; *Ho*: observed heterozygosity; *He*: expected heterozygosity; *F*_IS_: inbreeding coefficient.

**Fig 6:**
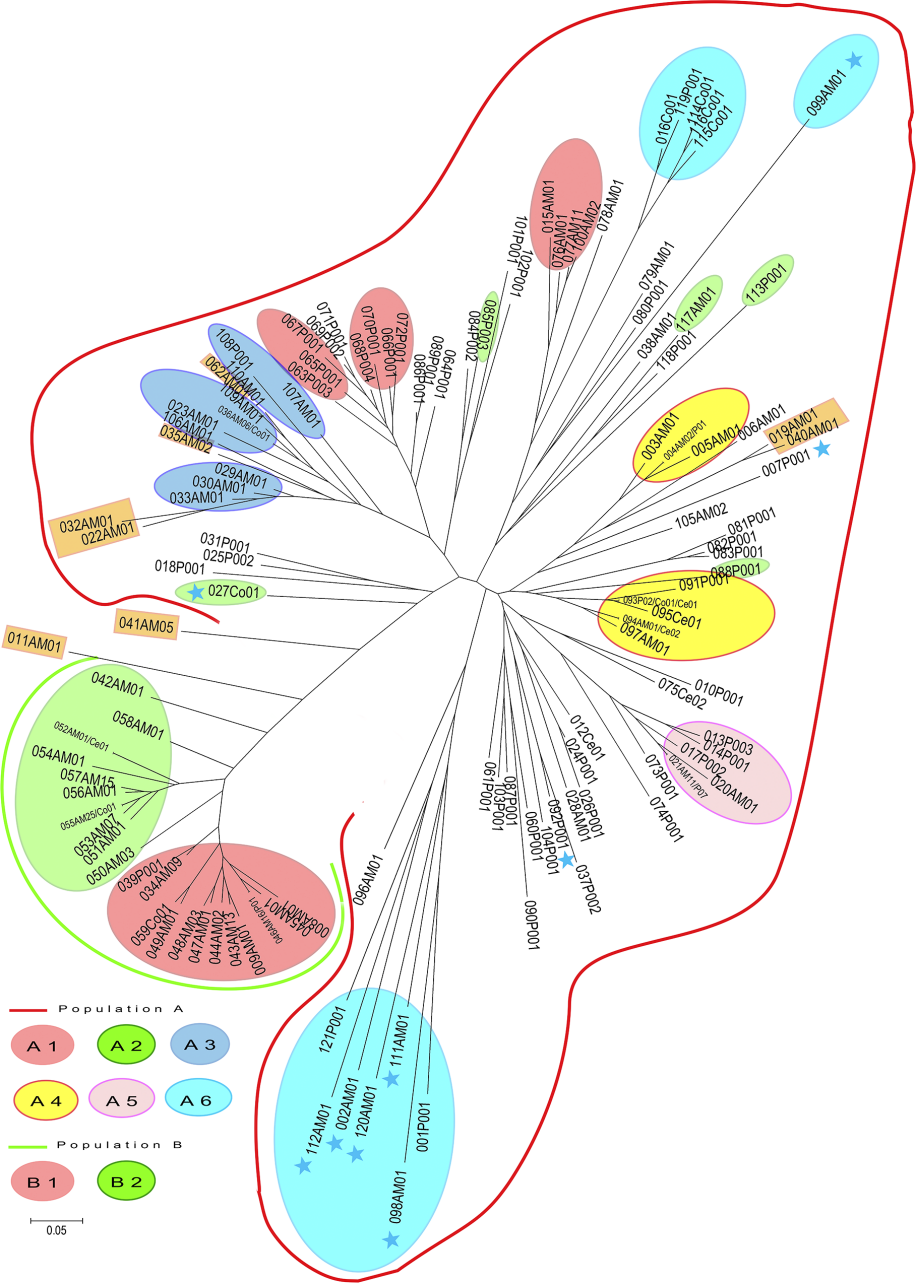
Unrooted neighbor-joining tree inferred from genetic distances derived from the proportion of alleles shared among the 270 isolates of *L*. *infantum* based on 12 microsatellite markers. The two populations defined using STRUCTURE are highlighted. The blue stars correspond to the non-MON-1 strains. At the end of each branche of the network, the first three characters correspond to the arbitrarily assigned number for each genotype, the following two characters (AM, P, Ce, Co) correspond to the endemic area of sample collection, and then the last two characters correspond to the number of isolates with the given genotype. The orange rectangles represent the isolates with mixed genotypes. The sub populations are represented with colored ovals at K = 6 for A sub-populations and K = 2 for B sub-populations. The isolates with no color are those with mixed genotypes within A sub-populations at K = 6. MEGA 4 software was used to visualize the neighbor-joining tree.

The NJ tree presented in Fig 6 provides a graphic representation of the data. The bootstrap values based on the re-sampling of loci were low and therefore not included in the NJ tree (Fig 6). This was due to the presence of admixed genotypes and the high number of shared alleles even if the allelic frequencies are different between the populations and sub-populations. Two main clusters were found to correspond to the two populations obtained using STRUCTURE at K = 2. Population B formed a separate cluster from Population A. The two sub-clusters defined using STRUCTURE for Populations B1 and B2 are shown on the NJ tree. However, for Population A, the clusters defined by the NJ tree did not perfectly correlate with the A sub-populations defined using STRUCTURE for neither K = 4 nor K = 6. The sub-populations A1, A2 and A3 (A1: four genotypes from AM and seven genotypes from P; A2: one genotype from AM, one from CO and three from P; and A3: eight genotypes from AM, two from P and one from CO) are dispersed throughout the NJ tree. Within Population A, the isolates from AM and P are dispersed throughout the cluster with no correlation with endemic area, clinical form or host background. The mixed genotypes were present between the two main clusters of Populations A and B as well as within the cluster of A sub-populations. Some non-MON-1 isolates grouped together as a paraphyletic group in the sub-population A6, while the others were dispersed among the MON-1 isolates and are present at the end of the branches. As previously described, the MON-108 isolate is closely related to the MON-1 isolates [23–25]. Regarding the nine isolates from AC, two (genotype 21) were present in sub-population A5, whereas seven (genotype 43) belonged to sub-population B1 and grouped with isolates from infant VL and HIV + VL patients. None of the isolates from AC has a mixed genotype. Samples isolated from CanL grouped together with human isolates, and no correlation was found between host and MLMT profile.

## Discussion

Leishmaniasis due to *L*. *infantum* is endemic in Southern France. In this study, we used MLMT, a molecular tool useful for population genetic studies, to analyze an extensive set of isolates from four endemic areas in Southern France (AM, P, CE and CO). To the best of our knowledge, this study is the first to investigate a large number of *L*. *infantum* isolates from different endemic areas in Southern France. We also focused on the AM and P endemic areas over an extended period of time. A greater number of samples came from AM than P (AM = 178 versus P = 75) because AM is the most active foci in France with the greatest number of leishmaniasis cases per year [2]. The study period was also longer for the AM area than for the P endemic area (AM: 1978–2011; P: 1993–2009). This aspect may generate a sampling bias, although no significant genetic differentiation was found when comparing isolates from AM and P during the same time period. Although MON-1 is the most prevalent zymodeme, other zymodemes also circulate in the south of France [7]. Microsatellite characterization of *L*. *infantum* isolates revealed a total of 121 different genotypes. Overall, 91 unique genotypes and 30 repeated genotypes were found. A greater number of repeated genotypes were observed in AM compared with P for the same period, thereby suggesting variations in the transmission cycle between the two areas such as outbreak, vector diversity or density, or host density.

In the AM endemic area, the isolates belonged to two main populations as defined by STRUCTURE: Population A and Population B. Within Population A, gene flow occurred between the AM and P endemic areas. The spread of isolates seems to be from AM to P, as indicated by the results of genotype 21, which was found 15 years later in P.

Substantial genetic diversity was found to be comparable to other endemic areas, even within zymodeme MON-1, thereby confirming previous analyses assessed by other markers [26]. As genetic differentiation depends on the area, our findings suggest strong epidemiological structuring. This is in agreement with the known mechanism of *Leishmania* transmission and spread in micro-foci and the entomologic data that have demonstrated limited sandfly dispersion [27–31]. Indeed, considering the behavior of the phlebotomine sandfly, it seems less likely that the spread of isolates from AM to P is due to sandfly movement [32]. Unfortunately, due to the small sample size of the phlebotomine sandfly isolates, we cannot investigate the transmission between sandflies, humans and canine hosts in further detail. We suspect that people traveling with their infected dogs between the endemic areas plays a possible role in the etiology of these exchanges. This has been already described for the emergence of *L*. *infantum* in South America probably via Conquistadores infected dogs from Portugal [33]. More recently, an intercontinental transportation from France to French Guiana was also reported due to the probable importation of *L*. *infantum* from an infected dog [34]. Notably, some repeated genotypes in the AM endemic area are well settled and continue to spread through the area over time. Indeed, some repeated genotypes were detected during a limited period, ranging from 6 to 15 years, and are no longer detected (genotypes 36, 43, 46, 53, 68 and 77). However, other genotypes were still detected in 2011 (genotypes 34, 55 and 57) and one genotype spread to P endemic area (genotype 21). The mixed genotypes between Population A and Population B were isolated in the AM endemic area, whereas in the A sub-populations, 75% of the mixed genotypes came from the P endemic area. We also observed a predominance of isolates from HIV+ patients in Population A (44.7%) compared with Population B (21.6%), which may indicate a variation in virulence. Indeed, these isolates from HIV+ patients may produce leishmaniasis in immunocompromised patients, whereas affected immunocompetent patients may develop only an asymptomatic infection [35]. This hypothesis is in agreement with the distribution of isolates from AC in both Population A and Population B. These isolates belonged to the genotypes 21 (2 AC) and 43 (7 AC), with samples from HIV+ VL, IVL, VL and CanL cases and samples from infant VL and HIV+ VL patients, respectively. The comparison of isolates from AC and those from HIV+ patients isolated during the same period and within the same restricted endemic area revealed no genetic differentiation between these populations. This finding contrasts with previous data reported by Hide et al. [9] and as suspected, does not reflect a difference in virulence. However, further isolates from asymptomatic carriers must be assessed to confirm our hypotheses. This is critical in the endemic areas of Southern France (as well as all endemic foci of leishmaniasis), where the isolates responsible for leishmaniasis represent only the tip of the iceberg [36]. Indeed, depending on the test used to detect asymptomatic carriage, prevalence varies from 30% to 46.8% in the AM endemic area [36]. Although our study provides important insight into leishmaniasis epidemiology in AM and P, our panel represents only a small proportion of the *L*. *infantum* population circulating in Southern France as samples from asymptomatic carriers, dogs and sandflies are underrepresented.

Microsatellite analyses may be useful to estimate relapse and re-infection rates, which is important to evaluate anti-*Leishmania* drug efficacy and transmission dynamics, respectively. This aspect is particularly important for the follow-up of patient treatment. MLMT may also be a useful tool to differentiate between relapses from re-infection cases [11,25,26,35,37,38]. Moreover, Bourgeois et al. have described “active chronic visceral leishmaniasis” in patients with several episodes of VL [20]. In this particular form of the disease, identifying the MLMT profile of each isolate responsible of each episode of VL could be useful to monitor and optimize treatment regimes. In our study, we detected probable treatment failure in HIV+ and non-HIV patients, as the MLMT profiles were indistinguishable from one episode to another. Certain patients likely experienced re-infection, as isolates from two different episodes of leishmaniasis in same patient displayed different MLMT profiles. However, we cannot exclude the possibility of a mixed infection with differential strain isolation depending on the time of sampling. Due to the small number of patients with isolates from several biological samples at different times, the rate of relapse and re-infection needs to be confirmed on a larger sample set. Thus, the results on relapse and re-infection should be interpreted with caution. Further investigations are required to assess these hypotheses in further detail.

The high ratio of repeated genotypes in HIV patients (81.6%) compared with the remaining population (41%) may be due to an outbreak amongst this fragile human population. Outbreaks have already been reported among intravenous drug users, a population also frequently affected by HIV infection [39,40]. Nevertheless, we have no information concerning this aspect of the case population in our study.

Faucher et al. have highlighted the heterogeneity of environments associated with VL transmission in Southeastern France [5]. The authors showed two distinct foci strongly associated with specific environments. One focus, corresponding to the AM endemic area, was characterized by scattered habitation and mixed forest in the foothills. In contrast, the other focus in the P endemic area was centered in urban areas of Marseille. These environmental differences correlate with the strong genetic differentiation we found between the *Leishmania* populations from AM and P. Indeed, the ecosystem influences the transmission cycle and thus the population dynamics of parasites. Moreover, in the P endemic area, Toscana virus, which is responsible for summer meningitis, and *L*. *infantum* share the same vector, *Phlebotomus perniciosus*. A recent study has described dogs co-infected by these two organisms [41]. Although cases of co-infection in humans or vectors have not been reported, we suspect that is also possible. This phenomenon of co-infection may have an impact on *Leishmania* transmission and should be addressed in future studies to understand whether this may also influence parasite evolution.

Other wild reservoirs of *L*. *infantum* have been demonstrated in Europe such as fox, rats and hare [42]. These wild reservoirs are able to transmit *L*. *infantum* to sandflies. However, the isolates from wild reservoirs have indistinguishable genotypes from those derived from domestic dogs and humans [26,43]. No isolates from wild animals were included in our study. The only uncommon host included in our study was a cat, and the isolate from this animal shared the genotype 55 with isolates from dog, IVL, HIV+ VL and VL samples.

In some studies, the isolates with a zymodeme other than MON-1 grouped together either via neighbor joining tree or STRUCTURE analysis [25,26]. We did not find such correlations with our data which is probably due to the high number of strains with mixed genotypes. Indeed the nine non-MON-1 isolates did not group into a separate population but rather clustered into Population A with a majority of the MON-1 isolates. In the NJ tree, some non-MON-1 isolates appeared as a paraphyletic group, while others were either isolated or dispersed among other zymodemes [23–25]. The zymodeme MON-108 (genotype 92) isolate appeared very close to MON-1 isolates with the genotype 37 [23–25].

In our study, no correlation was found between MLMT profile, clinical expression of the disease, immune status and host. Finally, MLMT is more discriminant and thus more appropriate than MLEE to evaluate epidemiological changes among parasite population in Southern France. MLMT data provided a better understanding of gene flow between *L*. *infantum* populations within the Southeastern France endemic area.

## Supporting Information

S1 Table Designation, characteristics and MLMT profiles of the *Leishmania infantum* isolates used in this study. AM: Alpes-Maritimes; P: Provence; CE: Cévennes; CO: Corsica. VL–Visceral leishmaniasis; IVL–Infant under 15 years visceral leishmaniasis; CL–Cutaneous leishmaniasis; MCL–Muco-cutaneous leishmaniasis; RVL—New episode of leishmaniasis in patients; AC–Asymptomatic carrier; CanL–Canine leishmaniasis; CatL–Leishmaniasis in cat; PHLE: isolate from phlebotomine sandfly; UK–Unknown. n. d. = not defined.
(XLS)
